# Knowledge, Attitude, and Practice of E-Cigarettes of Adolescents and Adults in Saudi Arabia: A Cross-Sectional Study

**DOI:** 10.3390/healthcare11222998

**Published:** 2023-11-20

**Authors:** Rasha Doumi, Sahar Khaytan, Alanoud Suliman Alobaidan, Bashayer Mohammad Alqahtany, Norah Mohammed Aldosari, Aljohara Ayed Almutairi, Alaa Askar Alanazi, Amel Fayed

**Affiliations:** College of Medicine, Princess Nourah Bint Abdulrahman University, Riyadh 11671, Saudi Arabia; 437000375@pnu.edu.sa (S.K.); 438001486@pnu.edu.sa (A.S.A.); 436004174@pnu.edu.sa (B.M.A.); 438001566@pnu.edu.sa (N.M.A.); 437003448@pnu.edu.sa (A.A.A.); 436007357@pnu.edu.sa (A.A.A.); aafayed@pnu.edu.sa (A.F.)

**Keywords:** electronic cigarettes, e-cigarettes, vaping, nicotine, youth, electronic nicotine-delivery systems (ENDS), Saudi Arabia

## Abstract

E-cigarettes have gained enormous popularity, and their use has increased drastically worldwide. However, little is known regarding adolescents’ and adults’ knowledge, attitudes, and practices in Saudi Arabia. We conducted a cross-sectional study using a self-administered online-modified WHO GATS questionnaire on a convenience sample approach. Data were collected between January and March 2021 after the alleviation of COVID-19 lockdown measures in Saudi Arabia. Univariate and multivariate regression models were developed to identify independent factors associated with knowledge, attitude, and practice. Our sample (1335) had a mean age of 26.45 ± 10.5 years; nearly half of the participants had poor knowledge about e-cigarettes. The usage and positive attitude were reported by 18.6% and 19.4%, respectively. Around 43.5% of e-cigarette users reported starting or increased use during the COVID-19 pandemic, while 9.5% of participants would recommend it to others. Logistic regressions showed that older participants were more likely to have poor knowledge (OR = 1.02, 95% C.I. = 1.01–1.03) and positive attitudes (OR = 0.98, 95% C.I. = 0.91–0.96). Male participants and smokers (OR = 3.0, 95% C.I. = 2.3–3.8) were more likely to have a positive attitude. However, younger participants were less likely to go for e-cigarettes (OR = 0.95, 95% C.I. = 0.93–0.97), while males (OR = 2.53, 95% C.I. = 1.65–3.86) and smokers (OR = 4.63, 95% C.I. = 3.47–6.18) were more likely to use them. This study indicated a high level of poor knowledge about e-cigarettes. A considerable proportion of participants reported usage and a positive attitude towards them. Older age, male gender, and being a smoker were the main elicited predictors for e-cigarette use.

## 1. Introduction

Electronic cigarette smoking is becoming popular among the youth, thus raising many concerns [1,2]. E-cigarettes (ECs) or electronic nicotine-delivery systems (ENDS) are battery-operated apparatus that transmit nicotine by the inhalation of vapor. E-liquid solutions almost always include nicotine, flavoring, and a humectant to retain moisture and create the aerosol when heated [3]. The use of these e-cigarettes is known as “vaping”, which makes the lungs more vulnerable to infections and weakens the immune system [4].

The practice of e-smoking is excessively increasing [5]. It is found that 4–6 percent and 8–10 percent of smokers have used ECs in the UK and the U.S., respectively [6]. An increase in the initiation of EC smoking is noticed among young adults and non-smokers [6]. Another European study reported an increase in adults who have tried ECs by 21% between the years 2014 and 2017 [7], in contrast with South Korea, where the usage of ECs was 9.2% in adolescents in 2011 [8]. Three studies conducted in Saudi Arabia showed that 28.9–49.4% of their participants use it to quit traditional smoking [9,10,11].

This increase in EC use could be due to multiple factors, such as its usefulness in smoking cessation, diminishing cigarette utilization, abating tobacco longing, and relieving stress when utilized as a substitute for normal cigarettes, in addition to saving money due to the high prices of conventional cigarettes [11,12].

Other factors relevant to young adults are peer pressure and upgraded taste and scent compared to conventional cigarettes. ECs do not have the strong lingering smell of traditional cigarettes, making them more acceptable in social settings. An examination of tobacco industry documents outlines the perceived benefits of flavored products to consumers, including pleasing aromas and aftertaste, increased excitement about the flavors and smoking enjoyment, and a “high curiosity to try” factor [13]. The COVID-19 pandemic was possibly a related factor affecting the usage of heated tobacco products and electronic cigarettes, as shown by an Italian study’s results in 2022, where 1.8% of ECs non-users began using them during the lockdown [14].

ECs are considered less harmful and less addictive than traditional cigarettes by many users. About 30% of surveyed Jordanian university students thought that smoking ECs was less risky than smoking regular cigarettes [15], with more than half of the respondents (56.3%) in a Malaysian study reporting ECs as being less harmful than conventional cigarettes to be their main reason to use it [16]. This is concerning with the debate that labels may not reflect the actual content of nicotine in ECs [4,16]. A study in Poland showed significantly higher nicotine dependence levels among exclusive EC users compared to traditional tobacco smokers [17]. ECs that contain nicotine, a well-known addictive substance, can cause chronic obstructive lung disease [18]. Nicotine in ECs improves mood and concentration, decreases anger and stress, and relaxes muscles. These provide a pleasant and good mood that might make youth addicted to smoking ECs. Furthermore, the liquid in ECs, e-liquid, includes a range of other potentially hazardous substances that are toxic to cells and may cause lung toxins or inflammatory obstruction of the lung’s smallest airways, known as popcorn lung disease [12]. Nicotine can negatively affect the brain and nervous system development in children and adolescents with resultant long-term consequences [4,19]. The WHO has stated that adolescents using ECs can have an increased risk of smoking cigarettes [4]. Secondhand EC exposure was also thought to be related to mental health problems [20]. The availability and access to ECs and social media promotions had the main effect on nicotine-containing ECs use among the youth [21]. This is of special concern with a recent local study highlighting the use of ECs to be significantly higher among young adults [22], consistent with internationally revealed data [23]. Among adolescents, there are two contrasting theoretical models of EC use [13]. One model suggests that youth who try ECs have more conventional and health-oriented values [13]. Their choice of ECs would be motivated more by health concerns, so they would not find cigarettes or other substances attractive [13]. An alternative model suggests that e-cigarettes may appeal to youth by providing a means of rebelling against conventional values and engaging in behaviors that provide pleasant physical sensations and increase a positive mood [13].

The EC's use and related knowledge and attitudes gained more importance after the COVID-19 pandemic, as recent studies suggest a variable trend with a possible increased use [24,25]. This can be attributed to the pandemic-associated stress and social distancing issues that peaked nationally and internationally during the implemented lockdown measures nationally and internationally [25].

There is a lack of data regarding adolescents’ and adults’ knowledge, attitudes, and practices of ECs in Saudi Arabia. Most conducted studies focused on students or a specific region. Assessing the level of knowledge, attitude, and practice of ECs among the general population is essential, especially during the COVID-19 pandemic when youth are put under great stress and may resort to ECs for comfort. The purpose of this study is to explore the knowledge, attitudes, and practices of electronic cigarette use in Saudi Arabia just after the COVID-19 lockdown measures were lifted.

## 2. Materials and Methods

This was a cross-sectional study that was implemented from January to March 2021. It included all the regions of Saudi Arabia. Females, males, adults, and adolescents from different backgrounds were included. The inclusion criteria were to be an educated adult above 18 years old, read the questionnaire, and have smartphone access, with no gender or residency area restrictions.

Data were collected through a self-administered online questionnaire. The questionnaire (available as Supplementary Materials) was designed based on the World Health Organization (WHO) Global Adult Tobacco Survey (GATS) [26] with modifications to suit e-cigarette use and the addition of some questions to fit our study objectives. It included variables related to the participants’ knowledge, attitude, and practice of ECs and their sociodemographic characteristics. Certain questions related to the use during the COVID-19 lockdown were added to explore habit changes during the pandemic. The age groups were divided arbitrarily to have a balanced percentage in the two categories. The participant’s level of education was classified as a school (up to 12 years of essential education), university (Bachelor), and postgraduate (Master and PhD).

The sample size was calculated using the G-power program, where the expected prevalence of EC smoking was 20% (10% margin of error), the level of confidence was 95% (alpha = 0.05), and the power of the study was 95%, so the minimal sample size needed for this study was estimated to be 808. We increased the sample size to 1335 to compensate for any incomplete responses and to obtain a better representation of our convenient sample. The REDcap web application [27] was used for making the surveys and collecting the participants’ responses, where a convenience sampling strategy was used to invite potential participants via various social media platforms (WhatsApp groups and Snapchat posts). We reached out to the potential participants once to avoid the possibility of encountering survey fatigue. Data were entered directly into the REDcap database and were analyzed with the Statistical Package for Social Sciences “SPSS” version 25 (SPSS Inc., Chicago, IL, USA).

The outcome variable was the EC's knowledge, attitude, and practice. The exposure variables were the sociodemographic variables. A comprehensive knowledge scale consisting of 14 questions was used to evaluate the participants' knowledge. The participants’ responses were recorded as true, false, and I don’t know answers, then recoded as correct/incorrect, where the I don’t know responses were considered incorrect answers. Reverse coding was used for four questions of the knowledge scale (1, 3, 8, and 12). The items consisted of information pertaining to ECs, their utility, harms, and benefits. The level of knowledge was interpreted based on a cutoff point of <8 points out of 14, defined as poor knowledge. The attitude items were assessed on a five-point Likert scale where the participants’ responses were recorded as strongly agree, agree, neutral, disagree, and strongly disagree. Reverse coding was performed for question number 4 of the attitude scale. The items were rated with observed scores ranging from 0 to 60 (60 representing a negative attitude, the cutoff point at 60% (<36 points classified as a positive attitude)).

The questionnaire’s face validity was tested by consulting three subject experts who commented on the content validity, and their corrections were considered before piloting the questionnaire. The questionnaire was designed in English; then, bilingual speakers translated the tool from English to Arabic. A backward translation was conducted to clarify any language errors and confirm its clarity. A piloting of the questionnaire with a sample of 20 participants to evaluate its clarity and acceptability was performed, followed by further modifications. The reliability of the used sub-scales was evaluated by calculating the Cronbach’s alpha coefficient. According to our findings for the two used subscales, Cronbach’s alpha for the knowledge scale was 0.802, and for the attitude scale, it was 0.887, which indicates excellent internal consistency.

Descriptive statistics in terms of frequencies and percentages, means, and standard deviations were used to describe the criteria of the studied sample. Analysis of quantitative data using a t-test and association of qualitative variables using a chi-square test was conducted. A *p*-value less than 0.05 was considered statistically significant. Logistic regression models were adopted to identify the independent factors affecting the knowledge, attitude, and practice level among the studied sample.

## 3. Results

The sample studied was comprised of 1335 participants, of whom 57.1% were females. Their mean age was 26.45 ± 10.5, with most of the participants aged 24 years or less (58.4%). Most had a university degree (58.4%), and around a quarter were smokers (25.1%). Only about 18.6% of them were currently using e-cigarettes, while 45.6% had a family member or a relative who uses e-cigarettes. The study population has heard of ECs mostly through friends (60.4%) and social media (56.3%). The participants thought that the main reasons for starting or experiencing an increased e-cigarette use frequency during the pandemic period were reduced activity and entertainment purposes (5.0% and 3.6%, respectively), while the main reasons for stopping or experiencing a decreased use were the fear of COVID-19 complications and the preference to use it with friends (1.0% and 1.0%, respectively) (Table 1).

**Figure 1:**
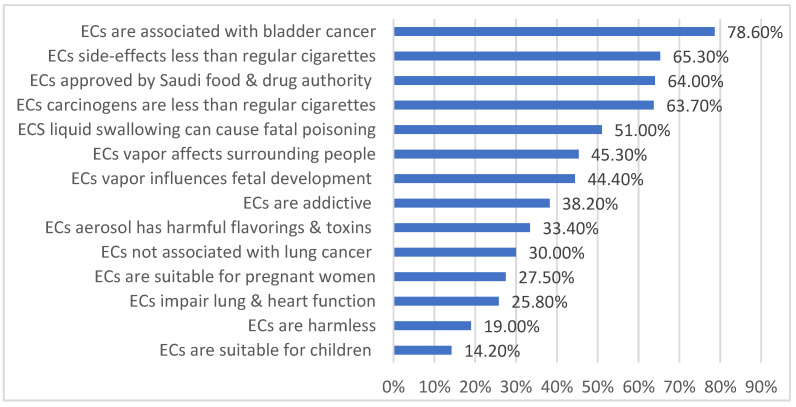
Frequency distribution of e-cigarette incorrect answers (poor knowledge) among study participants in KSA.

The mean knowledge score was 7.3 ± 2.8. About 48.9% of our sampled population exhibited poor knowledge levels. About half of the respondents (45.3%) falsely believed that ECs do not contribute to secondhand smoking. Around 78.6% did not know about ECs association with bladder cancer. Less than half of the respondents showed poor knowledge regarding e-cigarettes’ association with a number of health issues, including fetal development (44.4%), lung cancer (30%), and heart and lung function impairment (25.8%). More than a third of the participants could not correctly recognize its addictive effect (38.2%) (Figure 1).

**Figure 2:**
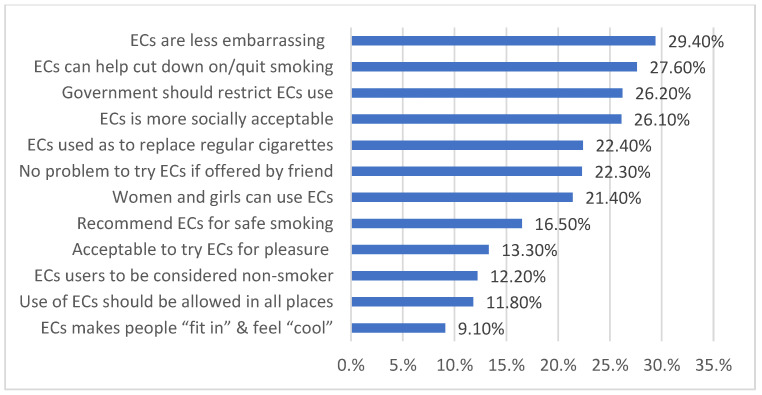
Distribution of positive attitudes towards e-cigarettes among study participants in KSA.

The total attitude score was 45.31 ± 10.77, with most participants showing a negative attitude (80.46%), while only 19.4% had a positive attitude. More than a quarter of the participants believed that e-cigarettes are not as embarrassing as regular cigarettes (29.4%) and can help people cut down on cigarettes or quit smoking (27.6%). Additionally, many participants thought that smoking ECs is socially acceptable (26.1%) and that the government should not restrict its use (26.2%). Some participants agreed that women and girls can use ECs (21.4%) and that it should be used as a replacement for regular cigarettes (22.4%). Around 22.3% had no problem trying ECs if offered by a friend (Figure 2).

We identified several factors that exhibited a significant association with EC knowledge, including age, gender, income, and smoking habits (*p* < 0.05). As shown in Table 2, participants aged 25 years or older reported increased levels of poor knowledge. Females (43.7%) and smokers (56.4%) showed significantly higher levels of poor knowledge. Participants with limited income were significantly more likely to have poor knowledge levels about e-cigarettes compared to those with higher incomes. There was a significant relation between age and attitude towards e-cigarette use, with younger people expressing a more positive attitude (*p* < 0.01). Also, males and school participants were significantly more likely to show a positive attitude (*p* < 0.01). Participants living in Riyadh were significantly less likely to show a positive attitude (*p* < 0.001) (Table 2).

The results showed a significant association between the use of ECs and several factors, including age, gender, education level, income, and smoking habits, as demonstrated in Table 2. Participants with a younger age of 24 years or less (22.3%) and males (32.7%) were significantly more likely to use e-cigarettes (*p* < 0.001). The highest proportion of EC use was reported among those with a school degree (21.9%). Current smokers and ex-smokers were more likely to use e-cigarettes (59.3% and 25.6%, respectively).

Among EC users, the most used flavors were fruit (51.6%) and mixed flavor (27.2%). Most users initiated its use for its taste (82.8%) or for entertainment effects (66.9%). The most reported side-effect was headaches, experienced by 39.7% of the EC users. Around 43.5% of EC users reported a start or an increase in its use during the COVID-19 pandemic. Only 9.5% would recommend using ECs to others (Table 3).

Three logistic regression models were developed to define the independent risk factors for poor knowledge and positive attitudes toward e-cigarettes and the use of e-cigarettes. For the poor knowledge level, only age was defined as an independent predictor for poor knowledge, with older participants more likely to have a poor level of knowledge (OR = 1.02, 95% confidence interval = 1.01–1.03). Regarding the positive attitude towards ECs, older participants were less likely to show a positive attitude towards ECs when compared to younger participants (OR = 0.98, 95% C.I. = 0.91–0.96). However, male participants were more likely to have a positive attitude as compared to females (OR = 1.7, 95% C.I. = 1.2–2.5), and smokers were three times more likely to have a positive attitude towards ECs (OR = 3.0, 95% C.I. = 2.3–3.8). Additionally, we tested the predictors for using ECs among our participants, and younger participants were less likely to go for ECs (OR = 0.95, 95% C.I. = 0.93–0.97), while male participants were more likely to use ECs as compared to females (OR = 2.53, 95% C.I. = 1.65–3.86), and current smokers had nearly four times the risk to use ECs as compared to non-smokers (OR = 4.63, 95% C.I. = 3.47–6.18) (Table 4).

## 4. Discussion

This study evaluated the knowledge, attitude, and practice of EC use among the study participants recruited from all the regions of Saudi Arabia and tested their possible correlation with sociodemographic factors. Our sample included participants of both genders and from different age groups with a recruited large sample size, which increased the representativeness of the target population. Our findings showed that nearly half of the participants had poor knowledge about ECs and their health hazards. The usage and the positive attitude towards ECs were reported by a considerable proportion, especially among males and younger ages. Alarmingly, 43.5% of e-cigarette users reported a start or an increase in its use during the COVID-19 pandemic, and unfortunately, 9.5% of all participants would recommend it to others. Older age, male gender, and being a smoker were the main elicited predictors for EC use.

Nearly half of our participants exhibited poor knowledge levels, comparable to a prior local study among undergraduate students [28] and lower than a Lebanese study conducted in 2020 [12], which was expected because of the different demographic and measurement tools. It was very alarming for us that a high proportion of our participants were unable to recognize the health hazards of ECs like bladder cancer, fetal development, lung cancer, and heart and lung function impairment. Also, more than a third of the participants could not correctly recognize the ECs' addictive effect, which was almost like another local study's findings [29]. Such findings were consistent across different local and regional studies [11,28,29,30] and can reflect a general issue in public awareness about ECs. Thus, these points should be highlighted in any further health education intervention. Only age was defined as an independent predictor for poor knowledge, where older participants were more likely to have a poor level of knowledge of ECs, highlighting an expected trend as the younger population was generally thought to be more exposed to knowledge and behaviors related to vaping and smoking from their peers and social media [31,32].

Most of our participants heard of ECs from their friends (60.4%) and social media (56.3%), which was in the same line as the findings of many prior studies [11,31,32]. This could be attributed to the known effect of friends and social media influencers on promoting different addiction habits. It is concerning, especially that more studies demonstrated the issue of vaping being positively addressed by influencers on various social media platforms, with much content promoting its use and not presenting the age and health-related warnings [32,33]. Around 22.3% of the participants had no problem trying ECs if offered by a friend.

Fortunately, our studied population displayed a negative attitude towards EC use. However, more than a quarter of the participants believed that ECs can help people cut down on regular cigarettes or quit smoking. This finding/trend was also reported from different regional countries but to different extents, ranging from 20.2 to 69.1% [2,12,34]. We reported that male participants and smokers were more likely to have a positive attitude towards ECs, which was consistent with a recent Malaysian study published in 2021 [34]. This raised the alarm to target these population groups in any further plans to improve their behavior. However, older participants were less likely to show a positive attitude towards ECs.

Different prevalences of using ECs were reported from different populations and regions nationally and internationally, with numbers as low as 5.1% and as high as 33.5% [12,29,35,36,37]. These differences were related to the different targeted populations and the geographical regions included in these studies (adolescent vs. adults, both genders, and even geographical regions). Our prevalence of 18.6% of EC usage falls within the range of these prior reported results. Younger participants were less likely to go for ECs, which contrasts the results of a European study on adolescents and young adults, where younger participants were the highest users [38]. In contrast, male participants and smokers were more likely to use them, which is consistent with several local, regional, and international studies [2,12,29,35,39,40,41]. The effect of gender in our community may be attributed to the nature of its conservative culture, where females rarely report smoking. The observed increase in the likelihood of smokers using e-cigarettes could be related to their possible trials to quit regular cigarette smoking or to other factors such as their expected addictive behavior, though, unfortunately, we did not assess their exact possible reasons in this study [2,3,8,42].

The behavior of people during pandemics and crisis situations was generally known to be different from their normal levels. Fortunately, our study was conducted in the second stage following the lockdown to prevent the spread of COVID-19 in the country, so many participants recall the events that happened during the lockdown. The pandemic had both positive and negative effects on EC use. The main reasons for the possible start or increase in EC use during that period were the reduced activity because of the lockdown and the entertainment purposes, while the fear of COVID-19 complications and the preference to use it with friends could explain stopping or experiencing a decreased EC use as thought by our participants and reported in the literature [43]. A significant proportion of EC users reported a start or increase in using the ECs during the COVID-19 pandemic, which was higher than reported in a previous US study [43]. On the other hand, our participants reported lower rates of stopping or decreasing the use of ECs during the pandemic than in a prior UK study [44].

This study’s limitations include the use of a convenience sampling strategy, as it can affect the representativeness of the target population, which we tried to limit by increasing the number of recruited participants. Also, collecting the data through online self-administrated questionnaires raises the possibility of reporting errors. However, the online distribution of the questionnaire helped us to reach a higher number of populations from different regions of Saudi Arabia.

Our results highlighted a gap in knowledge and attitudes related to ECs and issues related to their use among adolescents and young adults in Saudi Arabia. These can be used to inform targeted future health education programs and preventive interventions. Future research is recommended to further investigate the underlying factors leading to the increasing use of ECs in Saudi Arabia and test measures to reduce them. Holding nationwide awareness campaigns could be useful for increasing knowledge levels, popularizing more negative attitudes, and encouraging the public not to use ECs.

## 5. Conclusions

According to our results, data indicated a high level of poor knowledge about e-cigarettes. A considerable proportion of participants reported usage and a positive attitude towards it. Alarmingly, nearly half of the e-cigarette users reported a start or an increase in its use during the COVID-19 pandemic. Older age, male gender, and being a smoker were the main elicited predictors for e-cigarette use.

## Figures and Tables

**Table 1:** Sociodemographic profile and smoking habits distribution of our study participants in KSA.

Variables	N (%)
**Age groups (n = 1268)**	
24 years or less	741 (58.4%)
25 years or more	527 (41.6%)
**Gender (n = 1334)**	
Female	762 (57.1%)
Male	572 (42.9%)
**Education (n = 1333)**	
School	462 (34.7%)
University degree	779 (58.4%)
Postgraduate studies	92 (6.9%)
**Income (n = 1334)**	
Enough	637 (47.8%)
Enough and save	508 (38.1%)
Not enough	117 (8.8)
Not enough and barrow	72 (5.4%)
**Residency (n = 1335)**	
Riyadh	872 (65.3%)
Outside Riyadh	463 (34.7%)
**Smoking habits (n = 1333)**	
Currently smoking	335 (25.1%)
Non-smokers	920 (69.0%)
Ex-smokers	78 (5.9%)
**Participants currently using e-cigarettes**	248 (18.6%)
Sources of knowledge about e-cigarettes *	
Friends	857 (60.4%)
Social media	798 (56.3%)
Family	248 (17.5%)
Center of smoking cessation	60 (4.2%)
**Have a family member or a relative who uses e-cigarettes (n = 1308)**	597 (45.6%)

* Not mutually exclusive variable.

**Table 2:** Differences in sociodemographic variables among study participants with poor knowledge, positive attitude towards e-cigarettes, and e-cigarette use in KSA.

Variables	Poor Knowledge		Positive Attitude		E-Cigarette Use	
	N (%)	*p*-Value	N (%)	*p*-Value	N (%)	*p*-Value
**Age**						
24 years or less	298 (42.3%)	*p* < 0.01	178 (25.0%)	*p* < 0.001	165 (22.3%)	*p* < 0.001
25 years or more	255 (52.0%)		63 (12.5%)		70 (13.3%)	
**Gender**						
Female	314 (43.7%)	*p* < 0.05	93 (12.8%)	*p* < 0.001	60 (7.9%)	*p* < 0.001
Male	268 (50.7%)		153 (28.2%)		187 (32.7%)	
**Education level**						
University	354 (48.8%)	*p* = 0.151	130 (17.4%)	*p* < 0.001	141(18.1%)	*p* < 0.01
School	185 (42.9%)		109 (25.0%)		101 (21.9%)	
Postgraduate	42 (46.7%)		7 (8.0%)		6 (6.5%)	
**Income**						
Not enough and barrow	32 (49.2%)	*p* < 0.05	5 (7.5%)	*p* < 0.05	8 (11.1%)	*p* < 0.05
Not enough	61 (55.0%)		28 (25.5%)		25 (21.4%)	
Enough and save	200 (41.2%)		100 (20.3%)		110 (21.7%)	
Enough	289 (49.4%)		113 (18.7%)		105 (16.5%)	
**Residency**						
Riyadh	366 (45.1%)	*p* = 0.126	141 (16.9%)	*p* < 0.01	149 (17.1%)	*p* = 0.052
Outside Riyadh	217 (49.7%)		106 (24.1%)		99 (21.4%)	
**Smoking habits**						
Smoker	176 (56.4%)	*p* < 0.001	128 (40.6%)	*p* < 0.001	198 (59.3%)	*p* < 0.001
Non-smokers	375 (43.7%)		90 (10.2%)		30 (3.3%)	
Ex-smokers	30 (40.0%)		29 (38.7%)		20 (25.6%)	

**Table 3:** Distribution of e-cigarette use habits/practices among study participants in KSA.

Variables	N (%)
**Used e-cigarette flavors (n = 246)**	
Mint flavors	3 (1.2%)
Fruit flavor	127 (51.6%)
Mixed flavor	67 (27.2%)
Coffee flavor	4 (1.6%)
No flavor	5 (2.0%)
Reasons to start using e-cigarettes *	
Better taste than regular cigarettes (n = 244)	202 (82.8%)
Social smoking (with friends or family) (n = 242)	114 (47.1%)
Feeling stressed (n = 241)	122 (50.6%)
Feeling depressed (n = 240)	106 (44.2%)
Peer pressure (n = 238)	47 (19.7%)
To quit smoking of regular cigarettes (n = 237)	130 (54.9%)
Believe it is healthier than regular cigarettes (n = 240)	147 (61.3%)
To follow the trend (n = 238)	30 (12.6%)
Entertainment effect (relaxant, etc.) (n = 236)	158 (66.9%)
Cheaper than regular cigarettes (n = 239)	89 (37.2%)
Prior experience of possible side-effects of e-cigarette use *	
Bad breath (n = 242)	24 (9.9%)
Change in gum (n = 241)	19 (7.9%)
Loss of teeth/Change in teeth color (n = 241)	19 (7.9%)
Change in voice (n = 240)	26 (10.8%)
Headaches (n = 242)	96 (39.7%)
**The e-cigarette smoking frequency during the COVID-19 pandemic (n = 246)**	
Start/increased frequency during the pandemic	107 (43.5%)
Same as before the pandemic	92 (37.4%)
Stop/decreased frequency during the pandemic	47 (19.1%)
**Participants would recommend the use of e-cigarettes to others (n = 1295)**	123 (9.5%)

* Not mutually exclusive variable.

**Table 4:** Values of adjusted odds ratios with confidence intervals for significant factors associated with knowledge, attitude, and practice among study participants in KSA.

Variables	Poor Knowledge		Positive Attitude		E-Cigarette Use	
	N (%)	Adj. OR (C.I.)	N (%)	Adj. OR (C.I.)	N (%)	Adj. OR (C.I.)
**Age**						
24 years or less	298 (42.3%)	1	178 (25.0%)	1	165 (22.3%)	1
25 years or more	255 (52.0%)	1.02, (1.01–1.03)	63 (12.5%)	0.98, (0.91–0.96)	70 (13.3%)	0.95, (0.93–0.97)
**Gender**						
Female	314 (43.7%)	1	93 (12.8%)	1	60 (7.9%)	1
Male	268 (50.7%)	0.79, (0.60–1.02)	153 (28.2%)	1.7, (1.2–2.5)	187 (32.7%)	2.53, (1.65–3.86)
**Smoking habits**						
Non-smokers	375 (43.7%)	1	90 (10.2%)	1	30 (3.3%)	1
Smokers	176 (56.4%)	0.93, (0.75–1.15)	128 (40.6%)	3.0, (2.3–3.8)	198 (59.3%)	4.63, (3.47–6.18)
Ex-smokers	30 (40.0%)		29 (38.7%)		20 (25.6%)	

## Data Availability

The data presented in this study are available upon request from the corresponding author. The data are available on the Princess Nourah bint Abdelrahman University’s Redcap system.

## Supplementary Materials

The following supporting information can be downloaded at https://www.mdpi.com/article/10.3390/healthcare11222998/s1, E-cigarettes manuscript Supplementary File; the E-cigarettes related knowledge, attitude & practice among adolescents and adults in Saudi Arabia in 2021-Study Questionnaire.
