# Supplementary material for: Knowledge, Attitude, and Practice of E-Cigarettes of Adolescents and Adults in Saudi Arabia: A Cross-Sectional Study
Source: Healthcare (Basel). 2023 Nov 20;11(22):2998. doi: 10.3390/healthcare11222998 (PMC10671544; doi:10.3390/healthcare11222998)
Supplement: Supplementary file 1 [file healthcare-11-02998-s001.zip › healthcare-2691071-supplementary.pdf]

E-cigarettes manuscript Supplementary File

E-cigarettes related knowledge, attitude & practice among adolescents and adults in Saudi Arabia in 2021- Study Questionnaire

Personal data:

1- **Age** (in years) [ ]

2- Gender
- A- Male
- B- Female

3- Level of education
- A- Postgraduate
- B- University
- C- School

4- Income
- A- Enough and exceed needs
- B- Enough
- C- Not enough
- D- Not enough and borrow

5- Where are you from?
- A- Riyadh
- B- Outside Riyadh
  please specify [ ]

6- Do you currently smoke?
- A- Yes
- B- No
- C- Quit/quitting smoking

7- Where have you heard of E-cigarettes?
- A- Friends
- B- TV
- C- Social media
- D- Family
- E- University/school
- F- Doctors
- G- Center for smoking cessation

8- Do you have a family member or relative who use E-cigarettes?
- A. Yes
- B. No

Knowledge:

9. What do you think about the following statements?

No.	Statements	True	False	I do not know
1)	E- cigarettes are approved by Saudi food and drug authority			
2)	Swallowing the liquid in E-cigarettes accidentally can cause poisoning that is potentially fatal			
3)	E-cigarettes are not associated with lung cancer			
4)	E-cigarettes are associated with bladder cancer			
5)	E-cigarettes impair lung and heart function			
6)	E-cigarettes vapor has an effect on fetal development			
7)	Harmful flavorings and toxins are found in E-cigarettes aerosol			
8)	E-cigarettes are harmless			
9)	E-cigarettes are addictive			
10)	E-cigarettes has less side-effects than regular cigarettes			
11)	E-cigarettes carcinogenic component is less than the regular cigarettes			
12)	E-cigarettes are suitable for pregnant women			
13)	E-cigarettes are suitable for children			
14)	E-cigarettes vapor affect people surrounding the smoker			

Attitude:

10. What do you think about the following statements?

No.	Statements	Strongly agree	Agree	Neutral	Disagree	Strongly disagree
1)	The use of e-cigarette should be allowed in all places					
2)	A person who uses E-cigarettes should be considered as a non-smoker					
3)	E-cigarettes should be recommended to a nonsmoker who wants to try safe smoking					
4)	The government should restrict the use of E-cigarettes					
5)	e-cigarettes are not embarrassing like regular smoking					
6)	Women and girls can use e-cigarettes					
7)	Smoking e-cigarette makes people “fit in” and feel “cool”					
8)	It is socially acceptable to smoke E-cigarettes, compared to traditional cigarettes					
9)	E-cigarettes should be used as a replacement for regular cigarettes					
10)	It is acceptable to experiment with E-cigarettes for pleasure					
11)	E-cigarettes can help people cut down on cigarettes or quit smoking					
12)	I have no problem to try e-cig if offered by a friend					

Practice:

11. Do you currently use E-cigarettes?

A. Yes
B. No

<u>If yes</u>,

1- How long have you been using E-cigarettes? [ ]

12. How frequently do use e- cigarettes?
    A- Daily
    B- Not daily
    C- Do not know

2- What percentage of nicotine do you usually use in your e-cigarettes?
    A- I don't know
    B- No nicotine
    C- 2%
    D- 5%
    E- Other, please specify [ ]

3- Which of the e-cigarette flavors do you use?
    A- Coffee flavor
    B- Mint flavor
    C- Fruit flavor
    D- Mixed flavors
    E- No flavor
    F- Other, please specify [ ]

4- What made you start using E-cigarettes?

	Reasons	Yes	No
A	Better taste than regular cigarettes		
B	Social smoking (with friends or family)		
C	Stress		
D	Depression		
E	Peer pressure		
F	To quit smoking of regular cigarettes		
G	Believe its healthier than regular cigarettes		
H	To follow a trend		
I	Entertainment effect (relaxant, etc..)		
J	Cheaper than regular cigarettes		

Other reason, please specify [ ]

5- Have you experienced any of the following symptoms after using the E-cigarettes?

	Symptoms	Yes	No
A.	Bad breath		
B.	Change in your gums		
C.	Loss of teeth/change in your teeth color		
D	Change in your voice		
E.	headaches		

6- How is your E-cigarettes use frequency during COVID-19 pandemic?
   - A- I started using e-cigarettes during the pandemic
   - B- Same as before the pandemic
   - C- Increased frequency during the pandemic
   - D- Decreased frequency during the pandemic
   - E- I did not use E-cigarettes during the pandemic

12. What do you think the reasons for start using/increase in frequency of e-cigarettes use during the COVID-19 pandemic?
   - A- Stress
   - B- Depression
   - C- Loneliness
   - D- Anxiety
   - E- Reduced activities
   - F- For entertainment purposes
   - F- Other, please specify

13. What do you think the reasons for stop using/decrease in frequency of e-cigarettes use during the COVID-19 pandemic?
   - A- Financial reasons
   - B- Fear of COVID-19 complications in case got infected
   - C- Avoid side effect
   - D- Prefer to use it with friends
   - G- Other, please specify

14. Would you ever promote or recommend the use of E-cigarettes to other people if you got the chance to?
   - A- Yes
   - B- No
   - C- I do not know
